# Gender disparities in opioid treatment progress in methadone versus counseling

**DOI:** 10.1186/s13011-021-00389-4

**Published:** 2021-06-23

**Authors:** Erick Guerrero, Hortensia Amaro, Yinfei Kong, Tenie Khachikian, Jeanne C. Marsh

**Affiliations:** 1 I-Lead Institute, Research to End Health Disparities Corp, 12300 Wilshire Blvd, Suite 210, Los Angeles, CA 90025 USA; 2 Herbert Wertheim College of Medicine and Robert Stempel College of Public Health and Social Work, Florida International University, 11200 SW 8th ST, AHC4, Miami, Florida 33199 USA; 3 College of Business and Economics, California State University Fullerton, 800 N. State College Blvd, Fullerton, CA 92831 USA; 4 Crown Family School of Social Work, Policy, and Practice, University of Chicago, 969 E. 60th Street, Chicago, IL 60637 USA

**Keywords:** Gender disparities, Treatment progress, Opioid use, MOUD, Counseling, Methadone, Latina

## Abstract

**Background:**

In the United States, the high dropout rate (75%) in opioid use disorder (OUD) treatment among women and racial/ethnic minorities calls for understanding factors that contribute to making progress in treatment. Whereas counseling and medication for OUD (MOUD, e.g. methadone, buprenorphine, naltrexone) is considered the gold standard of care in substance use disorder (SUD) treatment, many individuals with OUD receive either counseling or methadone-only services. This study evaluates gender disparities in treatment plan progress in methadone- compared to counseling-based programs in one of the largest SUD treatment systems in the United States.

**Methods:**

Multi-year and multi-level (treatment program and client-level) data were analyzed using the Integrated Substance Abuse Treatment to Eliminate Disparities (iSATed) dataset collected in Los Angeles County, California. The sample consisted of 4 waves: 2011 (66 SUD programs, 1035 clients), 2013 (77 SUD programs, 3686 clients), 2015 (75 SUD programs, 4626 clients), and 2017 (69 SUD programs, 4106 clients). We conducted two multi-level negative binomial regressions, one per each outcome (1) making progress towards completing treatment plan, and (2) completing treatment plan. We included outpatient clients discharged on each of the years of the study (over 95% of all clients) and accounted for demographics, wave, homelessness and prior treatment episodes, as well as clients clustered within programs.

**Results:**

We detected gender differences in two treatment outcomes (progress and completion) considering two outpatient program service types (MOUD-methadone vs. counseling). Clients who received methadone vs. counseling had lower odds of completing their treatment plan (OR = 0.366; 95% CI = 0.163, 0.821). Female clients receiving methadone had lower odds of both making progress (OR = 0.668; 95% CI = 0.481, 0.929) and completing their treatment plan (OR = 0.666; 95% CI = 0.485, 0.916) compared to male clients and receiving counseling. Latina clients had lower odds of completing their treatment plan (OR = 0.617; 95% CI = 0.408, 0.934) compared with non-Latina clients.

**Conclusions:**

Clients receiving methadone, the most common and highly effective MOUD in reducing opioid use, were less likely to make progress towards or complete their treatment plan than those receiving counseling. Women, and in particular those identified as Latinas, were least likely to benefit from methadone-based programs. These findings have implications for health policy and program design that consider the need for comprehensive and culturally responsive services in methadone-based programs to improve outpatient treatment outcomes among women.

## Background

Individuals with opioid use disorders (OUD) report one of the highest dropout rates from substance use disorder (SUD) treatment [1–3]. The dropout rate in OUD treatment is on average 75% for the general population [4, 5], and higher among individuals identified as Black/African American [1, 6, 7], or Hispanic/Latino [8–11]. Despite the surge in opioid use among women [12] and their increasing access to OUD treatment [12, 13], there is limited knowledge about gender disparities in treatment progress in OUD treatment. Outpatient treatment for OUD is generally provided in two primary treatment service types: medication for OUD (MOUD, mainly methadone), and/or counseling [2, 9, 14]. Policy makers and public health experts have called for a systematic examination of the experience of women with OUD in different types of services (e.g., methadone- and counseling-based treatment) [15].

Most of the evidence on disparities in treatment outcomes based on gender, race or ethnicity has focused on SUD treatment in general instead of OUD treatment in particular. This growing literature offers some understanding of client and program factors that play a role in completing treatment [16, 17]. Client factors related to differential outcomes by gender are age, race/ethnicity, pregnancy status, and mental health status or symptoms [9, 10, 18, 19], whereas program factors that contribute to successful outcomes for women compared with men include treatment intensity [8, 19–21], retention in treatment [22–24], targeted services, and support for their roles as parents [25–27].

Although the literature on methadone treatment indicates its effectiveness in decreasing drug abuse, criminal behavior, and HIV risk [4, 13], and in increasing treatment retention [28] and counseling utilization [29–33], there has been limited evidence related to the impact of methadone on treatment progress and completion. Recent studies that examined all MOUD -not just methadone - found that these opioid medications were related to an increase in retention [28], but a decrease in treatment completion [1, 28]. Emerging work shows that methadone is associated with gender/race/ethnic disparities in retention in publicly funded OUD treatment [23]. However, there is little evidence of gender differences on the impact of methadone on treatment progress or completion.

A recent systematic review of gender disparities in treatment outcomes among people with OUD showed either no gender differences in treatment retention when receiving MOUD, or mixed findings [34]. For instance, when women received MOUD based on buprenorphine and naloxone, they were more likely than men to drop out. But when the MOUD was methadone, women were less likely to drop out than men [35]. Overall, we have limited information about gender disparities in OUD treatment progress towards meeting treatment plans based on program service type (methadone vs. counseling).

The publicly funded OUD treatment system in Los Angeles (LA), California (CA) is one of the largest and most diverse systems of care in the United States. This system has significantly expanded since publicly funded health insurance started covering treatment considering three program service types [36–38]: (1) specialty outpatient treatment programs offering MOUD, exclusively dispensing methadone; (2) outpatient services from treatment programs that mainly offer non-medication counseling services; and (3) office-based physician services offering MOUD and dispensing exclusively buprenorphine and or naltrexone. This publicly funded system mainly relies on (1) methadone, which may include limited counseling and (2) counseling-only [23]. These two service delivery types continue to expand in LA County following national trends [39, 40]. Unlike office-based services, counseling and methadone-based programs serve most (over 75%) opioid using clients in LA County, as well as most African American and Latino clients with OUD nationally [41–44].

It is unclear what the true delivery rates of methadone and counseling in OUD treatment are in Los Angeles County and nationwide. For instance, the Substance Abuse and Mental Health Service Administration (SAMHSA) treatment locator shows 100 adult outpatient SUD treatment programs in LA County. From this group, 55% of programs accept clients on MOUD prescribed elsewhere (i.e., some clients receive only counseling), 26% provide counseling and methadone and 4% do not use MOUD to treat OUD [45]. Client administrative data from the publicly funded system in LA County show that from 2011 to 2017, about 83% of discharged clients with OUD received methadone, while 17% received counseling only. It is not clear whether access to methadone has an equal impact on female and minority clients’ treatment progress, and/or likelihood to complete their treatment plan (e.g. reduce risk of opioid use and overdose).

It is critical to understand disparities in response to treatment in the most common type of OUD treatment where minority clients receive services (i.e., methadone or counseling). We draw from a healthcare disparities conceptual framework that identifies three stages to research disparities: (a) *detect* health care disparities in a vulnerable population; (b) *understand* client risk and program capacity factors; and (c) *reduce* disparities through provision of comprehensive services by high capacity programs [46]. We use the first two stages of the framework to (1) detect gender disparities in making progress towards or achieving completion of a treatment episode, and (2) understand which factors are associated with progress and completion for women and men with OUD when provided treatment based on methadone or counseling.

The research questions focus on the extent to which there are gender disparities in making progress towards and completing the treatment plan of clients who use opioids. The extant literature suggests that in general women, particularly women who identify as Black/African American or Latino/Hispanic, would have less progress and lower rates of completing treatment goals compared to men and clients identified as non-Latino White [1, 47, 48]. This literature suggests that individuals receiving methadone would make more progress or would be more likely to complete treatment goals than those receiving only counseling given that methadone-only treatment has proven effective and is the standard of care in OUD treatment [49, 50].

## Methods

### Data and Sample

We relied on client administrative data from the LA County Participant Reporting System (LACPRS) and integrated substance abuse treatment to end disparities (iSATed) Program Survey dataset [51]. The data came from a parent study funded by NIDA (R33 DA03563401) that focused on SUD treatment programs who served communities with more than 80% Latinos and or African American residents in LA County. We merged four waves of administrative client records with program survey data (2011, 2013, 2015, and 2017) and determined the sample of the current study using only programs serving clients with OUD in the parent study (see details elsewhere, [23]. These multi-year and multi-level (program- and client-level) cross-sectional data included 13,453 clients age 12 or older served by 135 unique SUD treatment “programs.” This sample included 34 (25.2%) SUD programs that offered outpatient counseling services to clients with OUD [no medication] and 101 (74.8%) outpatient programs that offered primarily methadone. These data do not provide information on whether clients attending methadone programs received additional services, such as counseling or ancillary services. These two types of programs services serve more than 95% of all clients entering publicly funded OUD treatment in L.A. County.

The sample consisted of the following samples per year/wave: 2011 (66 programs, 1035 clients: 358 female, 677 male), 2013 (77 programs, 3686 clients: 1076 female and 2610 male), 2015 (75 programs, 4626 clients: 1406 female and 3220 male) and 2017 (69 programs, 4106 clients: 1290 female and 2816 male). We only included cases that were discharged during each of the selected years (>95% of all clients) and that included counseling or methadone.

We relied on a random sample stratified by program service type resulting in an analytic sample of 80 SUD programs (20 counseling, and 60 methadone). Because fewer than 5% of clients receiving publicly funded treatment receive buprenorphine or naltrexone, we did not include those clients in the analytic sample. We focused on methadone, the most common OUD treatment publicly available [39] and one of the most cost-effective MOUDs [52].

## Measures

### Dependent Variables

We examined two dependent variables that reflected treatment outcomes at discharge: client made progress towards completing treatment/recovery plan and client completed treatment/recovery plan. These two outcomes relied on nine official discharge codes. The first two codes evaluated whether client completed the treatment/recovery plan, or were referred or transferred. The next two codes evaluated whether clients made significant progress towards completing their treatment/recovery plan, while the next five codes defined clients who left without making progress [53]. Our examination of gender disparities rely on discharged clients for any given year. For the first outcome, we coded 1 if the clinician reported the client was making progress towards completion, and 0 if not. For the second outcome, we coded 1 if the clinician reported the client completed the treatment/recovery plan for that episode, and 0 if not. These are measures that have been used to evaluate treatment completion in regional [54–56] and national studies [1, 16, 17, 28, 43]. They do not include information on the number, type or description of the treatment plan or its goals.

### Explanatory Variables

The independent variables of interest included clients’ self-reported sex, measured as a dichotomous variable (female = 1, male = 0). The study also examined race and ethnicity, in particular clients who identified as Latino/Hispanic, Black/African American, non-Latino White or Other. Clients who identified as non-Latino White were the reference category. We coded Latinos as a primary category and “other” as representing clients who identified as American Indian, Asian or Other, because our data did not have sufficient clients to analyze these groups separately. Clients also reported demographic variables including age, education, as well as homelessness and number of prior episodes in any alcohol or drug treatment/recovery program.

To create two mutually exclusive groups (counseling vs methadone) among discharged clients reporting opioid use, we selected OUD treatment programs and examined those that listed outpatient counseling (1 = Yes, 0 = No) and those that listed methadone (1 = Yes, 0 = No). We developed interaction terms using race/ethnicity, gender and program service type categories. For instance, we compared interaction terms for individuals who identify as Latino * female, with individuals identified as non-Latino White and males serving as the reference, and Latina * methadone with non-Latino White males and counseling as the reference.

### Analytical Approach

First, we conducted a comparative analysis based on clients receiving only methadone or only counseling. We relied on ANOVAs and Chi-Square tests to evaluate differences across the different individual characteristics of clients. We employed multilevel logistic regression to answer the main research question.

$$ logit\left(E(Y)\right)={\beta}_0+{\beta}_1\ast gender+{\beta}_2\ast treatment\ setting+{\beta}_3\ast year+{\beta}_4\ast race+{\gamma}_1\ast gender\times year+{\gamma}_2\ast gender\times treatment\ setting+{\gamma}_3\ast gender\times race+X\mathrm{B} $$

where *Y* refers to the binary dependent variables, making treatment progress or treatment completion, *X* denotes the vector of covariates, and Β is the coefficient vector for the covariates. The multi-level data structure, i.e. year-program-client, is accounted for by considering clients in the same program in the same year as a cluster. Correlation among those clients are incorporated when calculating the standard errors of coefficient estimates. We reported odd ratios considering the two binary outcomes. Finally, we also analyzed interactions such as gender and treatment type (e.g., female * methadone) using the same regression models mentioned above.

## Results

### Sample characteristics of clients that receive methadone versus counseling

Table 1 shows comparative statistics for opioid using clients who were discharged from outpatient methadone treatment (11,169 or 83%) or outpatient counseling treatment only (2384 or 17%). Clients in methadone-based programs reported lower rates than those in counseling in both progress towards completion (13% vs. 21% respectively) and completion (11% vs. 26% respectively). A lower percent of clients who made progress towards completing their treatment plan received methadone compared to counseling (13% vs. 21% respectively). Similarly, a lower percent of clients completing their treatment plan received methadone rather than counseling (10.7 vs. 26.1%). There were fewer females in the sample receiving methadone than receiving counseling (30% vs. 35% respectively).

**Table 1:** Comparative analysis of discharged clients with OUD by program service type (methadone and counseling)

	Methadone (*N* = 11169)	Counseling (*N* = 2284)
	Mean (SD) or Count (%)	Mean (SD) or Count (%)
Discharge status***		
Progress towards completing treatment plan	793 (12.7%)	279 (20.7%)
Completed treatment plan	668 (10.7%)	352 (26.1%)
No progress towards completing treatment plan	4765 (76.5%)	718 (53.2%)
Female***	3325 (29.8%)	805 (35.3%)
Wave		
2011***	490 (4.4%)	545 (23.9%)
2013***	3151 (28.2%)	535 (23.4%)
2015***	4043 (36.2%)	583 (25.5%)
2017***	3485 (31.2%)	621 (27.2%)
Race		
White***	4905 (44.2%)	1073 (48.1%)
African American***	1172 (10.6%)	163 (7.3%)
Latino**	4614 (41.5%)	850 (38.1%)
Other***	419 (3.8%)	146 (6.5%)
Age***	43.1 (13.4)	36.2 (11.8)
Education (years)	11.4 (2.9)	11.7 (2.9)
Homeless***	1447 (13.0%)	376 (16.5%)
# prior episodes***	2.7 (4.3)	3.2 (4.5)

*OUD* opioid use disorder, *SD* standard deviation

* *p* < 0.05; ** *p* < 0.01; *** *p* < 0.001

The results in Table 1 show that over the study period (2011–2017), more clients received methadone while the percentage of clients receiving counseling stayed relatively flat. The percentage of clients receiving methadone increased by an annual rate of 13.2% from 2011 to 2017, while the percentage of clients receiving counseling decreased by an annual rate of 11.9% over the same period. During the same period, the percentage of female clients receiving methadone increased by an annual rate of 14.2% while the percentage of female clients receiving counseling decreased by an annual rate of 11.4%. Meanwhile, the percentage of male clients receiving methadone increased by an annual rate of 12.7% while the percentage of male clients receiving counseling decreased by an annual rate of 12.1%.

Table 1 indicates racial/ethnic composition of clients in methadone versus counseling was significantly different at *P* < .05 (44.2% versus 48.1% for Whites; 10.6% versus 7.3% for African American; 41.5% versus 38.1% for Latino; and 3.8% versus 6.5% for Other). There were also significant differences in receiving either methadone or counseling (service type) across race/ethnic groups. Overall, clients who identified as White and those identified as Latino represented most clients in both methadone and counseling.

Younger clients were more likely to receive counseling than methadone (43 years with SD = 13.4 versus 36 years with SD = 11.8). Clients experiencing homelessness at intake also were more likely to be discharged from methadone (79% vs. counseling 21%). Individuals receiving counseling reported on average higher prior treatment episodes than individual receiving methadone (3.2 episodes with SD = 4.5 versus 2.7 episode with SD = 4.3).

## Gender differences in making progress or completing treatment plan

**Fig. 1:**
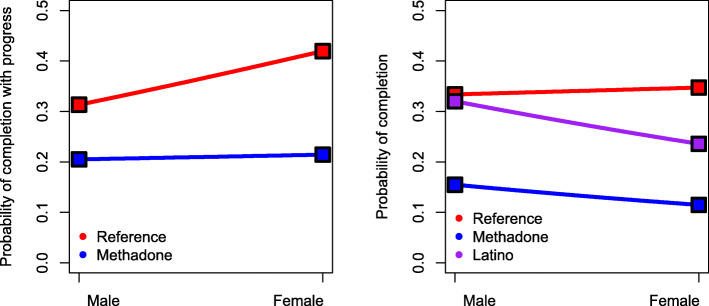
Moderated gender effects by program service type (methadone/counseling) and race/ethnicity. In model 1 (progress vs no progress presented in left figure), the relationship between gender and noncompletion with progress instead of without progress is moderated by methadone. The reference is clients receiving counseling only. In model 2 (completion versus noncompletion without progress presented in right figure), the relationship between gender and completion instead of noncompletion without progress is moderated by methadone and Latino. The reference is non-Latino White clients receiving counseling only

To examine gender differences in each of the two outcomes (making progress or completing treatment plan), we conducted three regression models on three mutually exclusive conditions (Model 1, progress/no progress, Model 2, completion/no progress and Model 3, completion/progress) as shown in Table 2. The following findings focus on Model 1 and 2 because we found no statistically significant relationships in Model 3, which considered clients who completed the treatment plan vs. clients who only made progress towards completing the treatment plan (*p* > .05). We present implications of these non-significant findings in the discussion section (see Table 2 and Fig. 1).

**Table 2:** Three logistic regressions based on two outcomes of treatment progress (Model 1) making **progress** and (Model 2 & 3) **completion** of treatment plan

	Model 1: Progress Vs No Progress			Model 2: Completion Vs No Progress			Model 3: Completion Vs Progress		
	OR	95% CI	*p* value	OR	95% CI	*p* value	OR	95% CI	*p* value
Female	**1.583**	**1.091, 2.298**	**0.016**	1.063	0.645, 1.752	0.812	0.693	0.479, 1.003	0.052
Year^a^									
2013	0.529	0.278, 1.007	0.052	**0.420**	**0.184, 0.957**	**0.039**	0.829	0.390, 1.759	0.625
2015	**0.330**	**0.178, 0.612**	**0.000**	**0.355**	**0.136, 0.930**	**0.035**	1.151	0.438, 3.021	0.776
2017	**0.294**	**0.146, 0.593**	**0.001**	0.334	0.103, 1.083	0.068	1.174	0.376, 3.671	0.782
Interactions (year * gender)									
2013 * Female	1.036	0.704, 1.525	0.858	1.533	0.901, 2.607	0.115	1.491	0.910, 2.444	0.113
2015 * Female	0.954	0.648, 1.404	0.810	1.524	0.888, 2.617	0.126	1.612	0.974, 2.668	0.063
2017 * Female	1.282	0.787, 2.088	0.318	1.222	0.747, 1.999	0.426	0.904	0.497, 1.642	0.740
Methadone^b^	0.565	0.302, 1.056	0.074	**0.366**	**0.163, 0.821**	**0.015**	0.604	0.243, 1.506	0.280
Interaction (treatment type * gender)									
Methadone * Female	**0.668**	**0.481, 0.929**	**0.016**	**0.666**	**0.485, 0.916**	**0.013**	1.017	0.727, 1.422	0.923
Race^c^									
Black	1.044	0.525, 2.074	0.903	0.659	0.297, 1.458	0.303	0.632	0.352, 1.132	0.123
Latino	0.852	0.627, 1.158	0.307	0.939	0.721, 1.225	0.645	1.174	0.930, 1.482	0.178
Others	1.019	0.648, 1.602	0.935	0.737	0.462, 1.176	0.201	0.671	0.396, 1.137	0.139
Interactions (race * gender)									
Black * Female	0.919	0.534, 1.582	0.760	0.666	0.357, 1.242	0.201	0.678	0.338, 1.358	0.273
Latino * Female	0.857	0.577, 1.272	0.444	**0.617**	**0.408, 0.934**	**0.023**	0.667	0.421, 1.056	0.084
Others * Female	0.670	0.312, 1.436	0.303	1.381	0.698, 2.734	0.354	1.967	0.813, 4.761	0.133
Age	1.009	0.999, 1.020	0.079	1.007	1.000, 1.014	0.052	0.999	0.988, 1.011	0.910
Education	1.009	0.979, 1.040	0.567	1.044	0.993, 1.098	0.090	1.040	0.996, 1.087	0.076
Homeless	**0.694**	**0.531, 0.908**	**0.008**	**0.681**	**0.516, 0.899**	**0.007**	1.037	0.717, 1.498	0.848
# prior treatment episodes	1.001	0.981, 1.020	0.955	1.002	0.976, 1.028	0.893	0.998	0.958, 1.039	0.910
# Observations used	6514			6467			2083		

Intragroup correlation among clients within the same program was accounted for by cluster() specification in stata

OR: odds ratio; CI: confidence interval

^a^year 2011 as reference

^b^Counseling as reference

^c^White as reference

In Model 1, we compared clients who made progress towards completing the treatment plan with clients who did not make progress. In Model 2, we compared clients who completed the treatment plan with clients who did not make progress towards completing treatment plan. We include below findings from interactions (gender, race/ethnicity and treatment type) as well.

Table 2 shows that in detecting disparities in progress towards treatment plan (Model 1), female clients were more likely to make progress towards completion than male clients (*OR* = 1.583; 95% CI = 1.091, 2.298). Compared to clients receiving services in 2011, clients receiving services in 2015 (*OR* = 0.330; 95% CI = 0.178, 0.612), and 2017 (*OR* = 0.294; 95% CI = 0.146, 0.593) had higher odds of making progress towards completing treatment plan. Findings from the interaction of gender and service type show that compared to males, female clients had lower odds of making progress towards completing the treatment plan in methadone compared with counseling and male clients (*OR* = 0.668; 95% CI = 0.481, 0.929).

Table 2 also shows that in detecting disparities in treatment completion (Model 2), there were no significant gender differences. But disparities were detected when considering gender, race/ethnicity and program service type. Clients receiving methadone had lower odds of completing their treatment plan (*OR* = 0.366; 95% CI = 0.163, 0.821). Findings based on race/ethnicity, gender and service type show that compared with males, female clients had lower odds of completing their treatment plan when receiving methadone versus counseling (*OR* = 0.666; 95% CI = 0.485, 0.916). Latinas had lower odds of completing their treatment plan compared to non-Latina clients (*OR* = 0.617; 95% CI = 0.408, 0.934). Those interaction are presented in Fig. 1.

Finally, other relevant relationships were identified based on year and homelessness. Compared to clients receiving services in 2011, clients receiving services in 2013 (*OR* = 0.420; 95% CI = 0.184, 0.957), and 2015 (*OR* = 0.355; 95% CI = 0.136, 0.930) had higher odds of completing the treatment plan. Clients reporting homelessness had lower odds of both making progress (*OR* = 0.694; 95% CI = 0.531, 0.908) and completing the treatment plan (*OR* = 0.681; 95% CI = 0.516, 0.899) compared to non-homeless clients.

## Discussion

We examined gender disparities in OUD treatment in two of the most common program service types available in the United States (methadone vs. counseling) [2, 9, 39]. In response to calls for a systematic examination of the experience of female clients in different types of OUD treatment service [15], this study draws from a healthcare disparities conceptual framework [46] to *detect* gender and race/ethnic disparities in making progress towards or completing treatment plan, and to *understand* factors associated with progress and completion for female and male clients with OUD when provided treatment based on counseling vs. methadone.

We did not *detect* gender disparities when we considered gender alone. Female clients had higher odds of making progress towards their treatment plan compared to male clients. However, when considering gender, race and service type, we *detected* disparities in both outcomes. In particular, Latinas had lower odds of completing their treatment plan at discharge compared to male clients and non-Latino white clients. These findings were consistent with other studies showing disparities in treatment engagement among female clients identified as Latinas [9, 10]. The most recent national study also shows that clients on methadone treatment were associated with decrease treatment completion of their treatment plan [28]. But other studies that consider methadone and buprenorphine have showed improved recovery outcomes compared to non-opioid replacement therapies, including drug-free counseling [57, 58]. Albeit conjectural, we believe that the observed difference in making progress is due to differences in the treatment experience of our low income minority sample of clients, and the potential differences in quality of care between counseling and methadone programs located in minority communities.

Some evidence suggests that low-income minority women are more likely to achieve their treatment/recovery goals when they receive more counseling sessions and comprehensive ancillary services (e.g., mental health, case management, child care) [10, 19, 20]. Outpatient methadone programs generally provide limited psychological and ancillary services compared to outpatient counseling-only programs [59]. These two program service types may also have different criteria for progress or completion of treatment plan. In counseling programs, progress and completion may be more about achieving drug-free recovery, whereas progress and completion in methadone programs may mean client does not relapse or overdose taking opioids other than methadone, like fentanyl.

The expansion of methadone treatment between 2011 and 2017 in one of the most populous and culturally diverse SUD treatment systems in the United States has implications for *understanding* differences in outcome based on program service type. In our large sample of programs in minority communities, both female and male clients in methadone-based programs had lower odds than those receiving counseling to complete their treatment plan. Although methadone is one the most cost-effective treatment for OUD [52], in this diverse and low-income community sample, female clients may have made more progress and completed their treatment goals because they received more ancillary support from counseling- vs. methadone-based programs.

Clients who were younger or experienced homelessness were more likely to receive counseling vs. methadone to treat their OUD. Similar to the factors described above affecting women in methadone, we believe that methadone program may pose individual and systemic barriers to certain vulnerable populations that need comprehensive services or that are not able to comply with methadone daily dosage delivered onsite.

Overall, findings *detecting* gender disparities in treatment plan progress and completion and *understanding* gender and service type as factors associated with these outcomes have implications for delivering gender-competent services, particularly in methadone programs. Because more than 75% of OUD clients in publicly funded treatment in LA County receive methadone, it is critical to develop health policies and additional resources for methadone-based programs to meet the service needs of low-income women. Health policies that reimburse programs for case management, child-care services, as well as psychological services to address women’s high rates of interpersonal trauma and childcare and parenting-related stress are needed. Findings also have implications for promoting a culture of gender-specific or gender-sensitive treatment in methadone programs. Finally, following guidelines from the National Institute of Drug Abuse [60] that highlight the need to address the whole person versus just medication needs of individuals with OUD is critical.

### Limitations

Our findings should be considered in light of the limitations of the study. We recognize that the strength of the administrative treatment data is the large and diverse sample of individuals with OUD and their status at treatment discharge. But these data and our approach have at least eight limitations that researchers should consider. First, our findings are limited to participation in the reported treatment program and consideration of only discharged clients within a year of entering OUD treatment, which represents most clients (>95%). Second, our data did not include information on the number or type of treatment plan goals so as to allow us to compare differences in treatment goals between patients in methadone compared to counseling. Third, the percentages of completion of treatment plan for clients with OUD in our LA County sample (18.4%) are below rates reported using the TEDS national data (27.8) [1]. Most completion studies using TEDS data use slightly different codes to develop the completion measure [16, 17, 28, 43, 54–56]. For instance, we included only outpatient clients whose primary or secondary drug of choice was opioids, who may have had previous treatment episodes and who were transferred to another program. A national study excluded clients whose secondary drug of choice was opioids, with previous treatment episodes and transferred to another program, and included both outpatient and residential treatment [1].

Albeit conjectural, we believe that these lower completion rates and the finding that women and minorities in methadone-based programs had worse outcomes than those who received counseling only is a result of our rigorous measure of completion and our sample of low-resourced programs in low-income minority communities. As methadone-based programs offer limited comprehensive services [59] that vulnerable populations require to make progress in OUD treatment, it is expected to see lower rate of progress and completion. The most recent findings on completion using national data are consistent with our results on decreased completion in (MOUD, methadone) versus non MOUD (e.g., outpatient) SUD treatment [1, 28].

A fourth limitation was not knowing how much counseling clients on methadone received or determining whether clients in counseling received any MOUD, including methadone, elsewhere. To mitigate the risk of crossover groups, we relied on auxiliary variables such as whether the client is using medications to improve the categorization of methadone vs. counseling. The resulting number of programs is consistent with the numbers reported in the SAMHSA provider locator (100 Adult outpatient SUD programs, from which 55% accept clients using MOUDs prescribed elsewhere, while 26% specifically rely on counseling [45]. A fifth limitation is that program closure and mergers did not allow us to have a longitudinal dataset to evaluate program effects overtime. Over 30% of programs closed at each wave, resulting in only 38 programs participating across our four waves of data. However, we controlled for the unbalanced and multi-level data and analyzed a sample that represented the SUD treatment system in minority communities at different years. A sixth limitation was that the multi-level data structure was only accounted for by considering the variance-covariance matrix for the sample in the same program. The mixed-effect model is preferred but the model-fitting process did not converge for our data. A seventh limitation was the small effect sizes. The effect size of the interaction between program service type and gender in models 1 and 2 was −0.07, whereas the effect size of the interaction between Latino and gender after standardization was −0.11. Although not large, these effect sizes are common in health services research and demonstrate the heterogeneity and importance of race and gender and treatment type in our observed outcomes. Finally, findings can only generalize to publicly-funded SUD treatment programs accepting opioid-using clients in communities with large Latino and African American populations, but this criteria applies to more than 7 million residents in L.A. County.

### Conclusions

We found significant gender disparities in completing OUD treatment plans at discharge when receiving methadone vs. counseling. Latinas were the most vulnerable to disparities in both outcomes (making progress and completing treatment plan). Methadone has proven effective to treat OUD (e.g., increase engagement [28, 31, 49], reduce overdose and relapses [30, 52], yet female clients, and Latinas in particular, report less progress in Methadone-based programs compared to counseling-only treatment. Overall, these findings may be explained by the potential differences in minority women’s comprehensive service needs (mental health therapy, child care services, etc.), program treatment approaches (drug-free recovery or methadone maintenance), and quality of care (culturally and linguistically responsive care). It is critical to develop evidence-based and culturally responsive OUD treatment interventions [51, 60] that further address the significant challenges that SUD programs face to ensure women equally benefit from OUD treatment regardless of the service delivery type.

## Data Availability

The datasets used and/or analyzed during the current study are available from the corresponding author and with permission of the Los Angeles County Department of Public Health on reasonable request.

## Abbreviations

ANOVA: Analysis of Variance
CA: California
CI: Confidence Interval
LA: Los Angeles
MOUD: Medication for opioid use disorder
NSATSS: National Substance Abuse Treatment Services Survey
OR: Odd Ratio
OTPs: Opioid treatment programs
OUD: Opioid use disorder
SAMHSA: Substance Abuse and Mental Health Service Administration
SUD: Substance use disorders
